# Supplementary material for: Severity of thermal burn injury is associated with systemic neutrophil activation
Source: Sci Rep. 2022 Jan 31;12:1654. doi: 10.1038/s41598-022-05768-w (PMC8803945; doi:10.1038/s41598-022-05768-w)
Supplement: Supplementary file 1 — Supplementary Information. [file 41598_2022_5768_MOESM1_ESM.docx]

**Supplemental Information**

**Severity of thermal burn injury is associated with systemic neutrophil activation**

Maria Laggner, Marie-Therese Lingitz, Dragan Copic, Martin Direder, Katharina Klas, Daniel Bormann, Alfred Gugerell, Bernhard Moser, Christine Radtke, Stefan Hacker, Michael Mildner, Hendrik Jan Ankersmit, Thomas Haider

# Supplemental Figure S1


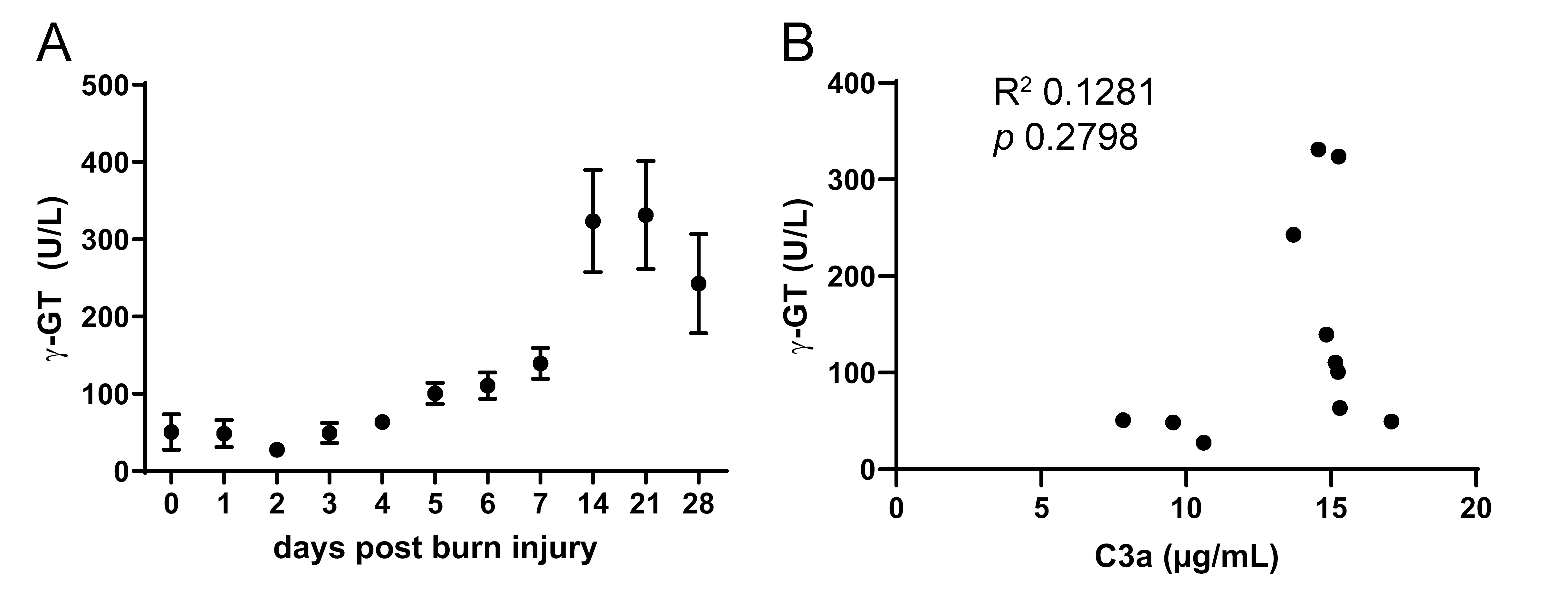


**Supplemental Figure S1. γ-GT levels following burn injury.** (A) Serum γ-GT levels in burn victims (n = 13-26) up to 4 weeks post injury. (B) Correlation between serum C3a and γ-GT concentrations over 28 days post burn trauma.

# Supplemental Figure S2

**Supplemental Figure S2. Lactadherin levels following burn injury.** Lactadherin concentrations in sera of burn victims (n = 5-6) and healthy controls (n = 2) over 4 weeks.

# Supplemental Figure S3

**Supplemental Figure S3. Correlations between serum levels of NETosis markers in burn victims.** Correlation between (A) MPO and NE, (B) CitH3 and NE, and (C) CitH3 and MPO on admission day.

# Supplemental Figure S4

**Supplemental Figure S4. NETosis levels in patients succumbing to burn injury.** Serum concentrations of neutrophil-derived factors were compared between survivors (n = 8-19) and deceased patients (n = 1-3).

# Supplemental Figure S5

**Supplemental Figure S5. Effect of inhalation trauma on serum NETosis levels.** Serum levels of neutrophil-derived factors were compared between burn victims with inhalation trauma (n = 1-3) and without inhalation trauma (n = 3-16). Serum levels of (A) MPO, (B) CitH3, (C) NE, and (D) C3a were assessed up to 4 weeks post burn injury.

# Supplemental Figure S6

**Supplemental Figure S6. Serum concentrations of neutrophil-derived factors in low and high APACHE II scores.** Scores were determined on admission day and categorized into low (≤18, n = 10) and high (≥19, n = 12) values. Serum concentrations of MPO, CitH3, NE, and C3a of the first 7 days post admission were compared. Data were compared by Mann-Whitney test.

# Supplemental Figure S7

**Supplemental Figure S7. Serum concentrations of neutrophil-derived factors in low and high SOFA scores.** Scores were determined on admission day and categorized into low (≤6, n = 15) and high (≥7, n = 16) values. Serum concentrations of MPO, CitH3, NE, and C3a of the first 7 days post admission were compared. Data were compared by Mann-Whitney test.

# Supplemental Figure S8

**Supplemental Figure S8. Immune cell counts of patients with higher and lower degree burns.** Data of 3^rd^ degree burns (n = 1-22) and lower degree burns (n = 1-10) were compared by Mann-Whitney test.

# Supplemental Figure S9

**Supplemental Figure S9. Immune cell counts of patients with high and low APACHE II scores.** Scores were determined on admission day and categorized into low (≤ 18, n = 1-10) and high (≥ 19, n = 1-12) values. Data were compared by Mann-Whitney test.

# Supplemental Figure S10

**Supplemental Figure S10. Immune cell counts of patients with high and low SOFA scores.** Scores were determined on admission day and categorized into low (≤ 6, n = 1-15) and high (≥ 7, n = 1-16) values. Data were compared by Mann-Whitney test.

# Supplemental Figure S11

**Supplemental Figure S11. Immune cell counts of patients with high and low ABSI scores.** Scores were determined on admission day and categorized into low (≤ 8, n = 1-19) and high (≥ 9, n = 1-13) values. Data were compared by Mann-Whitney test.
